# Prevalence of Chronic Pain and High-Impact Chronic Pain Among Adults — United States, 2016

**DOI:** 10.15585/mmwr.mm6736a2

**Published:** 2018-09-14

**Authors:** James Dahlhamer, Jacqueline Lucas, Carla Zelaya,, Richard Nahin, Sean Mackey, Lynn DeBar, Robert Kerns, Michael Von Korff, Linda Porter, Charles Helmick

**Affiliations:** ^1^Division of Health Interview Statistics, National Center for Health Statistics, CDC; ^2^National Center for Complementary and Integrative Health, National Institutes of Health, Bethesda, Maryland; ^3^Division of Pain Medicine, Stanford Medicine, Stanford, California; ^4^Kaiser Permanente Washington Health Research Institute, Seattle, Washington; ^5^Departments of Psychiatry, Neurology and Psychology, Yale University, New Haven, Connecticut; ^6^National Institute of Neurological Disorders and Stroke, National Institutes of Health, Bethesda, Maryland; ^7^Division of Population Health, National Center for Chronic Disease Prevention and Health Promotion, CDC.

Chronic pain, one of the most common reasons adults seek medical care (*1*), has been linked to restrictions in mobility and daily activities (*2*,*3*), dependence on opioids (*4*), anxiety and depression (*2*), and poor perceived health or reduced quality of life (*2*,*3*). Population-based estimates of chronic pain among U.S. adults range from 11% to 40% (*5*), with considerable population subgroup variation. As a result, the 2016 National Pain Strategy called for more precise prevalence estimates of chronic pain and high-impact chronic pain (i.e., chronic pain that frequently limits life or work activities) to reliably establish the prevalence of chronic pain and aid in the development and implementation of population-wide pain interventions (*5*). National estimates of high-impact chronic pain can help differentiate persons with limitations in major life domains, including work, social, recreational, and self-care activities from those who maintain normal life activities despite chronic pain, providing a better understanding of the population in need of pain services. To estimate the prevalence of chronic pain and high-impact chronic pain in the United States, CDC analyzed 2016 National Health Interview Survey (NHIS) data. An estimated 20.4% (50.0 million) of U.S. adults had chronic pain and 8.0% of U.S. adults (19.6 million) had high-impact chronic pain, with higher prevalences of both chronic pain and high-impact chronic pain reported among women, older adults, previously but not currently employed adults, adults living in poverty, adults with public health insurance, and rural residents. These findings could be used to target pain management interventions.

NHIS is a cross-sectional, in-person, household health survey of the civilian noninstitutionalized U.S. population, conducted by the National Center for Health Statistics (NCHS).* Data from the 2016 Sample Adult Core for adults aged ≥18 years (33,028; response rate = 54.3%)^†^ were analyzed. Information about pain was collected through responses to the following questions: “In the past six months, how often did you have pain? Would you say never, some days, most days, or every day?” and “Over the past six months, how often did pain limit your life or work activities? Would you say never, some days, most days, or every day?” Chronic pain was defined as pain on most days or every day in the past 6 months, as recommended by the International Association for the Study of Pain,^§^ modified to account for intermittent pain, and used in both the National Pain Strategy and National Institutes of Health Task Force on Chronic Back Pain (*6*). As suggested in the National Pain Strategy, high-impact chronic pain was defined as chronic pain that limited life or work activities on most days or every day during the past 6 months (*5*). The prevalence of chronic pain and high-impact chronic pain (both crude and age-adjusted, with 95% confidence intervals) were estimated for the U.S. adult population overall and by various sociodemographic characteristics. These characteristics, collected with the Family Core questionnaire, included age, sex, race/ethnicity, education level, current employment status,^¶^ poverty status (calculated using NHIS imputed income files),** veteran status, health insurance coverage type (reported separately for adults aged <65 and ≥65 years), and urbanicity. All prevalence estimates met NCHS reliability standards.^††^ Because pain prevalence varies by age, age-adjusted estimates were used in comparisons of chronic pain and high-impact chronic pain between subgroups. Based on two-tailed Z-tests, all reported differences between subgroups are statistically significant (unless otherwise noted; p<0.05). Analyses were conducted using statistical software that accounts for the stratification and clustering of households in the NHIS sampling design. Estimates incorporated the final sample adult weights adjusted for nonresponse and calibrated to population control totals to enable generalization to the civilian noninstitutionalized population aged ≥18 years.

In 2016, an estimated 20.4% of U.S. adults (50.0 million) had chronic pain and 8.0% of U.S. adults (19.6 million) had high-impact chronic pain (Table), with higher prevalence associated with advancing age. Age-adjusted prevalences of both chronic pain and high-impact chronic pain were significantly higher among women, adults who had worked previously but were not currently employed, adults living in or near poverty, and rural residents. In addition, the age-adjusted prevalences of chronic pain and high-impact chronic pain were significantly lower among adults with at least a bachelor’s degree compared with all other education levels.

**TABLE:** Prevalence of chronic pain* and high impact chronic pain^†^ among U.S. adults aged ≥18 years, by sociodemographic characteristics—National Health Interview Survey, 2016

Characteristic	Chronic pain*			High-impact chronic pain^†^		
	Estimated no.^§^	Crude % (95% CI)	Age-adjusted^¶ ^% (95% CI)	Estimated no.^§^	Crude % (95% CI)	Age-adjusted^¶ ^% (95% CI)
**Total**	**50,009,000**	**20.4 (19.7–21.0)**	**19.4 (18.7–20.0)**	**19,611,000**	**8.0 (7.6–8.4)**	**7.5 (7.1–7.9)**
**Age group (yrs)**						
18–24	2,082,000	7.0 (5.8–8.5)	—**	446,000	1.5 (0.9–2.3)	—**
25–44	11,042,000	13.2 (12.3–14.1)	—**	3,681,000	4.4 (3.9–5.0)	—**
45–64	23,269,000	27.8 (26.6–29.0)	—**	10,044,000	12.0 (11.2–12.9)	—**
65–84	11,808,000	27.6 (26.4–29.0)	—**	4,578,000	10.7 (9.9–11.6)	—**
≥85	1,766,000	33.6 (30.1–37.3)	—**	830,000	15.8 (13.2–18.9)	—**
**Sex**						
Male	21,989,000	18.6 (17.7–19.5)	17.8 (17.0–18.7)	8,276,000	7.0 (6.5–7.6)	6.7 (6.2–7.3)
Female	28,049,000	22.1 (21.2–23.0)	20.8 (19.9–21.6)	11,296,000	8.9 (8.4–9.4)	8.2 (7.7–8.7)
**Race/Ethnicity**						
Hispanic	5,856,000	15.1 (13.6–16.7)	16.7 (15.2–18.4)	2,754,000	7.1 (6.0–8.3)	7.9 (6.9–9.2)
White, non-Hispanic	36,226,000	23.0 (22.2–23.8)	21.0 (20.3–21.8)	13,230,000	8.4 (7.9–8.9)	7.4 (7.0–7.9)
Black, non-Hispanic	5,148,000	17.9 (16.4–19.6)	17.8 (16.3–19.4)	2,387,000	8.3 (7.2–9.4)	8.1 (7.1–9.2)
Other, non-Hispanic^††^	2,774,000	13.8 (12.1–15.7)	14.4 (12.7–16.3)	1,326,000	6.6 (5.3–8.1)	7.0 (5.7–8.5)
**Education**						
Less than high school	7,809,000	26.1 (24.2–28.2)	23.7 (21.7–25.7)	4,069,000	13.6 (12.3–15.2)	12.1 (10.7–13.7)
High school/GED	14,441,000	23.7 (22.5–25.0)	22.6 (21.2–23.9)	5,910,000	9.7 (9.0–10.6)	9.1 (8.4–10.0)
Some college	17,129,000	22.6 (21.5–23.8)	22.9 (21.8–24.0)	6,518,000	8.6 (7.9–9.4)	8.7 (8.0–9.5)
Bachelor's degree or higher	10,383,000	13.4 (12.6–14.3)	12.4 (11.7–13.3)	2,944,000	3.8 (3.4–4.3)	3.5 (3.1–4.0)
**Employment status**						
Employed	22,085,000	14.7 (14.1–15.5)	14.5 (13.8–15.2)	5,108,000	3.4 (3.1–3.8)	3.2 (2.9–3.6)
Not employed; worked previously	25,737,000	31.5 (30.3–32.7)	29.2 (27.8–30.6)	13,318,000	16.3 (15.4–17.2)	16.1 (15.0–17.3)
Not employed; never worked	2,083,000	15.9 (13.8–18.2)	18.7 (16.1–21.6)	1,192,000	9.1 (7.6–10.9)	11.1 (9.1–13.4)
**Poverty status**						
<100% FPL	8,017,000	25.8 (24.2–27.6)	29.6 (27.9–31.3)	4,630,000	14.9 (13.6–16.4)	17.5 (16.1–19.0)
100% ≤FPL<200%	11,357,000	26.2 (24.5–27.9)	25.9 (24.2–27.7)	5,375,000	12.4 (11.3–13.6)	12.3 (11.2–13.5)
200% ≤FPL<400%	14,181,000	20.3 (19.2–21.4)	19.3 (18.3–20.4)	5,100,000	7.3 (6.7–8.1)	6.9 (6.2–7.6)
≥400% FPL	16,441,000	16.3 (15.4–17.2)	14.6 (13.8–15.5)	4,438,000	4.4 (4.0–4.9)	3.9 (3.5–4.4)
**Veteran**						
Yes	6,379,000	29.1 (27.1–31.2)	26.0 (23.5–28.7)	2,258,000	10.3 (9.1–11.8)	9.2 (7.7–11.1)
No	43,519,000	19.5 (18.9–20.2)	19.0 (18.4–19.7)	17,407,000	7.8 (7.4–8.2)	7.5 (7.1–7.9)
**Health insurance coverage^§§^**						
Age <65 yrs						
Private	20,539,000	15.1 (14.3–15.8)	14.0 (13.3–14.8)	5,713,000	4.2 (3.8–4.7)	3.8 (3.4–4.2)
Medicaid and other public coverage	8,215,000	29.3 (27.3–31.5)	30.0 (28.0–32.2)	4,822,000	17.2 (15.6–19.0)	17.8 (16.2–19.6)
Other	3,860,000	43.5 (40.0–47.2)	34.8 (31.2–38.7)	2,263,000	25.5 (22.5–28.8)	19.3 (16.4–22.5)
Uninsured	3,683,000	16.2 (14.4–18.2)	17.0 (15.2–19.0)	1,319,000	5.8 (4.7–7.2)	6.2 (5.0–7.6)
Age ≥65 yrs						
Private	5,606,000	28.0 (26.3–29.9)	28.1 (26.3–30.0)	1,842,000	9.2 (8.1–10.5)	9.3 (8.2–10.6)
Medicare and Medicaid	1,428,000	42.5 (37.6–47.5)	42.5 (37.6–47.5)	816,000	24.3 (20.4–28.6)	24.3 (20.4–28.6)
Medicare Advantage	3,094,000	25.5 (23.1–28.1)	25.8 (23.4–28.4)	1,226,000	10.1 (8.5–11.8)	10.3 (8.7–12.1)
Medicare only, excluding Medicare Advantage	2,115,000	25.9 (23.1–28.9)	25.9 (23.1–28.9)	939,000	11.5 (9.5–13.7)	11.5 (9.5–13.7)
Other	1,229,000	31.6 (27.2–36.3)	31.8 (27.4–36.5)	545,000	14.0 (11.3–17.3)	14.3 (11.5–17.7)
Uninsured	106,000	—^¶¶^	—^¶¶^	59,000	—^¶¶^	—^¶¶^
Urbanicity***						
Urban	38,401,000	19.0 (18.3–19.7)	18.4 (17.7–19.0)	14,754,000	7.3 (6.9–7.8)	7.0 (6.6–7.4)
Rural	11,575,000	26.9 (25.4–28.5)	24.0 (22.5–25.6)	4,776,000	11.1 (10.2–12.2)	9.8 (8.8–10.9)

**Abbreviations:** CI = confidence interval; FPL = federal poverty level; GED = General Educational Development certification.

* Pain on most days or every day in the past 6 months.

^†^ Chronic pain limiting life or work activities on most days or every day in the past 6 months.

^§^ The estimated numbers, rounded to 1,000s, were annualized based on the 2016 data. Counts for adults of unknown status (responses coded as “refused,” “don’t know,” or “not ascertained”) with respect to chronic pain and high-impact chronic pain are not shown separately in the table, nor are they included in the calculation of percentages (as part of either the denominator or the numerator), to provide a more straightforward presentation of the data.

^¶^ Estimates are age-adjusted using the projected 2000 U.S. population as the standard population and five age groups: 18–29, 30–39, 40–49, 50–59, and ≥60 years.

** Not applicable.

^††^ Non-Hispanic other includes non-Hispanic American Indian and Alaska Native only, non-Hispanic Asian only, non-Hispanic Native Hawaiian and Pacific Islander only, and non-Hispanic multiple race.

^§§^ Based on a hierarchy of mutually exclusive categories. Adults reporting both private and Medicare Advantage coverage were assigned to the Medicare Advantage category. “Uninsured” includes adults who had no coverage as well as those who had only Indian Health Service coverage or had only a private plan that paid for one type of service such as accidents or dental care. “Other” comprises military health care including TRICARE, VA, and CHAMP-VA, and certain types of local and state governmental coverage, not including the Children’s Health Insurance Program.

^¶¶^ Estimates are considered unreliable according to the National Center for Health Statistics’ standards of reliability.

*** Based on U.S. Census Bureau definitions of urban and rural areas (https://www2.census.gov/geo/pdfs/reference/ua/Defining_Rural.pdf).

Whereas non-Hispanic white adults had a significantly higher age-adjusted prevalence of chronic pain than did all other racial and ethnic subgroups, no significant differences in high-impact chronic pain prevalence by race/ethnicity were observed. Similarly, the age-adjusted prevalence of chronic pain was significantly higher among veterans than among nonveterans, but no significant difference was observed in the prevalence of high-impact chronic pain.

Among adults aged <65 years, the age-adjusted prevalences of chronic pain and high-impact chronic pain were higher among those with Medicaid and other public health care coverage or other insurance (e.g., Veteran’s Administration, certain local and state government) than among adults with private insurance or those who were uninsured. Among adults aged ≥65 years, those with both Medicare and Medicaid had higher age-adjusted prevalences of chronic pain and high-impact chronic pain than did adults with all other types of coverage.

## Discussion

Pain is a component of many chronic conditions, and chronic pain is emerging as a health concern on its own, with negative consequences to individual persons, their families, and society as a whole (*4*,*5*). *Healthy People 2020* (https://www.healthypeople.gov/), the nation’s science-based health objectives, has a developmental objective to “decrease the prevalence of adults having high-impact chronic pain.” This analysis extends previous national studies of chronic pain prevalence by identifying adults with high-impact chronic pain. In 2016, approximately 20% of U.S. adults had chronic pain (approximately 50 million), and 8% of U.S. adults (approximately 20 million) had high-impact chronic pain. This estimate of high-impact chronic pain is similar to or slightly lower than estimates reported in the few studies that have looked at pain using a similar construct. For example, a recent study that used a measure of high-impact chronic pain similar to the one used in this study reported an estimate of 13.7% among a sample of U.S. adult health plan enrollees (*7*). Similarly, a 2001 study of adults from a region in Scotland found that 14.1% of survey participants reported significant chronic pain, and 6.3% reported severe chronic pain, and a 2001 study of Australian adults reported that 11.0% of men and 13.5% of women reported chronic pain that interfered, to some degree, with daily life activities (*3*,*8*). The results of subgroup analyses in the current study were consistent with findings in these studies (*3*,*8*) insofar as the prevalence of high-impact chronic pain was higher among women, adults who had achieved lower levels of education, and those who were not employed at the time of the survey, and was lower among adults with private health insurance compared with public and other types of coverage. In addition, high-impact chronic pain was also found to be higher among adults living in poverty and among rural residents.

Socioeconomic status appears to be a common factor in many of the subgroup differences in high-impact chronic pain prevalence reported here. Indicators of socioeconomic status such as education, poverty, and health insurance coverage have been determined to be associated with both general health status and the presence of specific health conditions (*9*) as well as with patients’ success in navigating the health care system (*9*). Identifying populations at risk is necessary to inform efforts for developing and targeting quality pain services.

The findings in this report are subject to at least five limitations. First, data are self-reported and subject to recall bias. Second, data are cross-sectional, precluding drawing causal inferences. This might be particularly relevant for socioeconomic status, which can be both a risk factor for and a consequence of chronic pain or high-impact chronic pain, or both. Third, no information is available on treatment for chronic pain to assess the prevalence of chronic pain and high-impact chronic pain among those with and without treatment. Fourth, NHIS excludes important populations, such as active duty military and residents of long-term care facilities or prisons. And finally, NHIS does not collect data on chronic pain or high-impact chronic pain in children. Despite these limitations, three strengths of this study are that it used a large, nationally representative data source to produce estimates of chronic pain and high-impact chronic pain across many demographic subgroups, it used standard broad definitions of pain that were not limited to one or more specific health conditions (e.g., headache or arthritis), and it used the standard case definition for high-impact chronic pain proposed by the National Pain Strategy.

Chronic pain contributes to an estimated $560 billion each year in direct medical costs, lost productivity, and disability programs (*4*). The National Pain Strategy, which is the first national effort to transform how the population burden of pain is perceived, assessed, and treated, recognizes the need for better data to inform action and calls for estimates of chronic pain and high-impact chronic pain in the general population (*5*). This report helps fulfill this objective and provides data to inform policymakers, clinicians, and researchers focused on pain care and prevention.

**Summary**

**What is already known about this topic?**

Chronic pain has been linked to numerous physical and mental conditions and contributes to high health care costs and lost productivity. A limited number of studies estimate that the prevalence of chronic pain ranges from 11% to 40%.

**What is added by this report?**

In 2016, an estimated 20.4% of U.S. adults had chronic pain and 8.0% of U.S. adults had high-impact chronic pain. Both were more prevalent among adults living in poverty, adults with less than a high school education, and adults with public health insurance.

**What are the implications for public health practice?**

This report helps fulfill a National Pain Strategy objective of producing more precise estimates of chronic pain and high-impact chronic pain.

## Footnotes

* https://www.cdc.gov/nchs/nhis/index.htm.

† The sample adult respondent is randomly selected from all adults aged ≥18 years in the family and answers for himself/herself (unless physically or mentally unable to do so, in which case a knowledgeable adult serves as a proxy respondent). Although interviews are conducted in respondents’ homes, follow-ups by telephone to complete missing sections are permissible. For more information, see the 2016 National Health Interview Survey Public Use Data Release: Survey Description Document (ftp://ftp.cdc.gov/pub/Health_Statistics/NCHS/Dataset_Documentation/NHIS/2016/srvydesc.pdf).

§ The International Association for the Study of Pain’s definitions of chronic pain can be found in the *Classification of Chronic Pain, Second Edition (Revised)*. https://www.iasp-pain.org/PublicationsNews/Content.aspx?ItemNumber=1673&navItemNumber=677.

¶ Based on responses to the following questions: “What was [person]/were you doing last week?” and “Have you ever held a job or worked at a business?” Based on the first question, adults who were “working for pay at a job or business,” “with a job or business but not at work” or “working, but not for pay, at a family-owned job or business” were classified as currently employed. Adults who were “looking for work” or “not working at a job or business and not looking for work” based on the first question and who subsequently answered “yes” to the second question were classified as “previously employed.” Adults who were “looking for work” or “not working at a job or business and not looking for work” based on the first question and who subsequently answered “no” to the second question were classified as “never employed.”

** Federal poverty levels are updated annually by the U.S. Census Bureau (https://aspe.hhs.gov/computations-2016-poverty-guidelines). Percentage of poverty relative to the federal poverty level is used to define poverty status, and is calculated, using NHIS imputed income files, as total family income divided by the family’s corresponding federal poverty level, and multiplied by 100.

†† https://www.cdc.gov/nchs/data/series/sr_02/sr02_175.pdf.
